# Age-Stratified Differences in Cardio–Reno–Metabolic Risk Profiles

**DOI:** 10.3390/geriatrics11010018

**Published:** 2026-02-11

**Authors:** Mihaela Simona Popoviciu, Timea Claudia Ghitea

**Affiliations:** 1 Department of Preclinical Disciplines, Faculty of Medicine and Pharmacy, University of Oradea, 1 Decembrie, 410028 Oradea, Romania; mihaela.popoviciu@didactic.uoradea.ro; 2 Department of Internal Medicine II, Diabetes Mellitus, Clinical County Emergency Hospital of Oradea, 410167 Oradea, Romania; 3 Pharmacy Department, Faculty of Medicine and Pharmacy, University of Oradea, 1 University Street, 410087 Oradea, Romania

**Keywords:** aging, cardio–renal risk, metabolic syndrome, insulin resistance, TyG index, vascular aging, renal function, estimated glomerular filtration rate, blood pressure, life-course approach

## Abstract

**Background:** Susception to cardio–reno–metabolic disorders increases markedly with age; however, the dominant contributors to risk may differ across the adult life course. While metabolic abnormalities often predominate at younger ages, vascular and renal alterations become more prominent in older populations. Understanding how these risk components reconfigure with aging may inform age-tailored prevention strategies. **Methods:** This cross-sectional observational study included 287 adults undergoing clinical and biochemical evaluation for cardio–metabolic risk. Participants were stratified into three age categories: <65 years (*n* = 175), 65–75 years (*n* = 84), and >75 years (*n* = 28). Anthropometric measurements, blood pressure, metabolic parameters, liver enzymes, inflammatory markers, and renal function indices were assessed. Insulin resistance was estimated using the triglyceride–glucose (TyG) index, and renal function was evaluated by estimated glomerular filtration rate (eGFR) and urinary albumin-to-creatinine ratio (UACR). Comparisons across age groups were performed using one-way analysis of variance (ANOVA). **Results:** Younger participants (<65 years) exhibited a predominantly metabolic risk profile, characterized by higher body mass index, waist circumference, fasting plasma glucose, triglycerides, and TyG index (all *p* < 0.05). In contrast, advancing age was associated with a progressive vascular–renal phenotype, including higher systolic blood pressure, lower diastolic blood pressure, and a marked decline in eGFR (*p* < 0.001). Liver enzymes decreased with age, while the FIB-4 index increased. UACR and C-reactive protein levels did not differ significantly between age groups. Despite these differences in individual risk markers, the composite risk category score was similar across age strata. **Conclusions:** Cardio–reno–metabolic risk profiles show distinct age-stratified patterns in dominant risk markers, with metabolic predominance more evident at younger ages and vascular–renal vulnerability more prominent in older adults. These findings support a life-course perspective on risk assessment and highlight the potential importance of early detection of vascular and microvascular risk in metabolically burdened younger individuals, prior to the development of overt renal dysfunction and advanced vascular aging.

## 1. Introduction

Cardio–metabolic and renal disorders are major contributors to global morbidity and mortality, with their burden increasing substantially with advancing age. Aging is associated with complex physiological changes that affect glucose metabolism, lipid handling, vascular structure, and renal function, ultimately leading to a higher prevalence of cardiovascular disease, chronic kidney disease, and their shared risk factors in older populations. However, the relative contribution of metabolic versus vascular–renal mechanisms to overall risk may vary across different stages of adulthood and aging [1,2,3].

In younger and middle-aged adults, cardio–metabolic risk is often dominated by obesity, insulin resistance, dyslipidemia, and impaired glucose regulation. These metabolic abnormalities promote endothelial dysfunction, low-grade inflammation, and early microvascular injury, processes that may precede clinically overt cardiovascular or renal disease by many years. As individuals age, cumulative exposure to metabolic stressors, together with age-related vascular remodeling and arterial stiffness, may shift the risk profile toward hemodynamic and renal vulnerability [4,5,6,7,8].

In older adults, particularly those over 75 years of age, vascular aging becomes a central determinant of adverse outcomes. Increased systolic blood pressure, reduced diastolic pressure, and declining renal function reflect progressive arterial stiffness and microvascular damage. These changes are closely linked to cardiovascular events, functional decline, and increased mortality in geriatric populations. Importantly, renal impairment often represents a late manifestation of long-standing cardio–metabolic and vascular injury, raising questions about the optimal timing for risk detection and preventive interventions [9,10,11].

Despite extensive evidence linking aging to increased cardio–renal risk, relatively few studies have examined how the dominant components of cardio–reno–metabolic risk shift across age categories, particularly in populations with a high burden of metabolic disorders. Understanding whether metabolic dysregulation predominates at younger ages while vascular–renal alterations emerge later may have important implications for age-tailored risk stratification and prevention strategies [12,13].

From a clinical perspective, this life-course approach suggests that identifying vascular and microvascular risk at earlier stages—when metabolic abnormalities are already present but before irreversible vascular or renal damage has occurred—could offer a critical window for intervention. However, data directly comparing metabolic, vascular, and renal markers across distinct age strata within the same cohort remain limited.

The aim of this study was to investigate age-related differences in cardio–reno–metabolic risk profiles by comparing anthropometric, metabolic, vascular, hepatic, inflammatory, and renal parameters across three age categories (<65 years, 65–75 years, and >75 years). By characterizing how risk patterns shift with advancing age, this study seeks to provide insight into the potential importance of early vascular risk detection in metabolically burdened younger adults.

## 2. Materials and Methods

### 2.1. Study Design and Population

This study was designed as a cross-sectional observational analysis conducted in adult patients undergoing clinical and biochemical evaluation for cardio–metabolic risk. A total of 287 participants were included and stratified according to age into three predefined categories: <65 years, 65–75 years, and >75 years.

**Figure 1:**
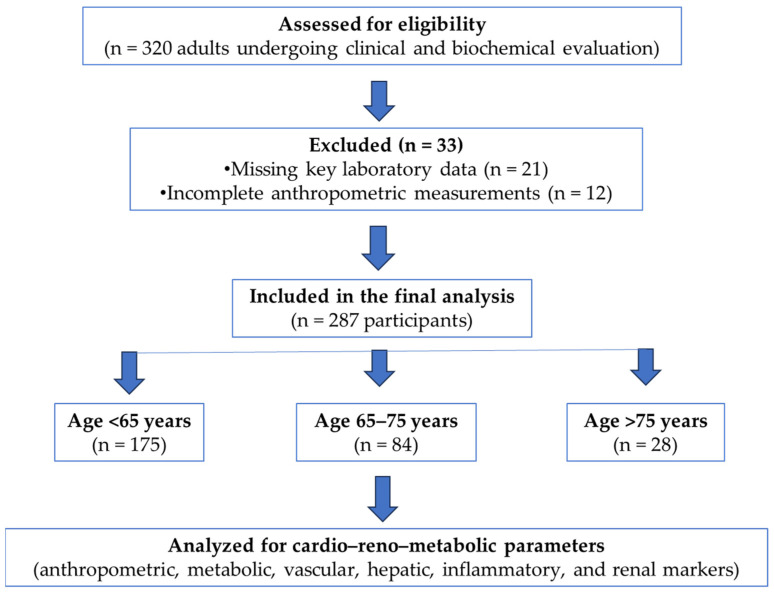
Flowchart.

Participants were included if complete clinical, anthropometric, and laboratory data were available at the time of assessment. Individuals with missing key variables required for the calculation of composite indices were excluded from the analysis (Figure 1).

### 2.2. Clinical and Anthropometric Assessment

Anthropometric measurements were performed using standardized procedures. Body mass index (BMI) was calculated as weight divided by height squared (kg/m^2^). Waist circumference was measured at the midpoint between the lower margin of the last palpable rib and the top of the iliac crest.

Blood pressure was measured in a seated position after a minimum of 5 min of rest, using a calibrated sphygmomanometer. Systolic (SBP) and diastolic blood pressure (DBP) values were recorded and used for analysis.

### 2.3. Laboratory Measurements

Venous blood samples were collected after an overnight fast. Laboratory analyses included investigations of the following:

- Fasting plasma glucose;
- Lipid profile, including triglycerides and HDL cholesterol;
- Liver enzymes, alanine aminotransferase (ALT) and aspartate aminotransferase (AST);
- Inflammatory markers and C-reactive protein (CRP);
- Hematological parameters, including platelet count;
- Renal function markers, serum creatinine and estimated glomerular filtration rate (eGFR);
- Urinary samples were collected for the assessment of urinary albumin-to-creatinine ratio (UACR).

The eGFR was calculated using the CKD-EPI creatinine equation and expressed as mL/min/1.73 m^2^.

### 2.4. Composite Indices

The triglyceride–glucose (TyG) index was calculated as a surrogate marker of insulin resistance using the established formula:

$$\mathrm{TyG}\;\mathrm{index}=ln\left(\frac{\mathrm{fasting}\;\mathrm{triglycerides}\;(\mathrm{mg}/\mathrm{dL})\times \mathrm{fasting}\;\mathrm{glucose}\;(\mathrm{mg}/\mathrm{dL})}{2}\right)$$

The FIB-4 index, a non-invasive marker of hepatic fibrosis risk, was calculated based on age, AST, ALT, and platelet count using the standard equation. Given that age is a component of the FIB-4 formula, this index was interpreted with caution in age-stratified analyses.

A composite risk category score was also recorded, reflecting global cardio–metabolic risk stratification according to institutional clinical protocols.

### 2.5. Data Collection

All personal identifiers were removed prior to analysis. Data were coded and stored on a password-protected institutional server accessible only to authorized investigators. Clinical and biochemical data were obtained by the same trained medical team under standardized institutional procedures. Anthropometric measurements were performed by certified nurses, and venous blood samples were collected by licensed laboratory staff.

### 2.6. Statistical Analysis

SPSS (v30) was used for data cleaning, visualization, and regression modeling with robust (HC3) standard errors, and for classical statistical tests (normality, ANOVA, and correlation analyses) to ensure consistency and cross-validation of results. Continuous variables were expressed as mean ± standard deviation (SD) or median (IQR), and categorical data as frequencies (%). Between-group comparisons across metabolic risk categories were performed using one-way ANOVA or the Kruskal–Wallis test, as appropriate. Correlations were evaluated using Pearson’s r and Spearman’s ρ coefficients.

Normality was assessed using Shapiro–Wilk tests. For skewed variables (e.g., triglycerides, fasting glucose, UACR, CRP), nonparametric Kruskal–Wallis tests were performed to verify robustness of group comparisons.

### 2.7. Ethical Considerations

The study was conducted in accordance with the Declaration of Helsinki and approved by the Institutional Ethics Committee of the University of Oradea (protocol CEFMF/1, 31 January 2023).

## 3. Results

### 3.1. Study Population Characteristics

A total of 287 participants were included in the analysis and stratified into three age categories: <65 years (*n* = 175), 65–75 years (*n* = 84), and >75 years (*n* = 28), presented in Table 1. Sex distribution did not differ significantly across age groups (male/female: <65 = 94/81; 65–75 = 41/43; >75 = 10/18; *p* = 0.195).

### 3.2. Anthropometric Profile

Significant differences were observed in anthropometric parameters across age categories. Both body mass index and waist circumference decreased progressively with advancing age, with the lowest values recorded in participants older than 75 years. Mean BMI declined from 35.54 ± 5.03 in individuals younger than 65 years to 35.04 ± 3.97 in those aged 65–75 years and to 32.71 ± 2.29 in the oldest group (*p* = 0.009). Similarly, waist circumference showed a comparable age-related pattern, decreasing from 111.93 ± 10.82 cm to 111.25 ± 8.65 cm and 105.79 ± 4.75 cm across the respective age categories (*p* = 0.009).

### 3.3. Glycemic and Lipid Profile

Markers of glucose metabolism and insulin resistance differed significantly across age categories. Fasting plasma glucose levels were highest in participants younger than 65 years and showed a progressive decline with advancing age, decreasing from 162.48 ± 62.58 mg/dL in the youngest group to 148.06 ± 44.51 mg/dL in those aged 65–75 years and to 133.00 ± 31.98 mg/dL in participants older than 75 years (*p* = 0.012).

**Figure 2:**
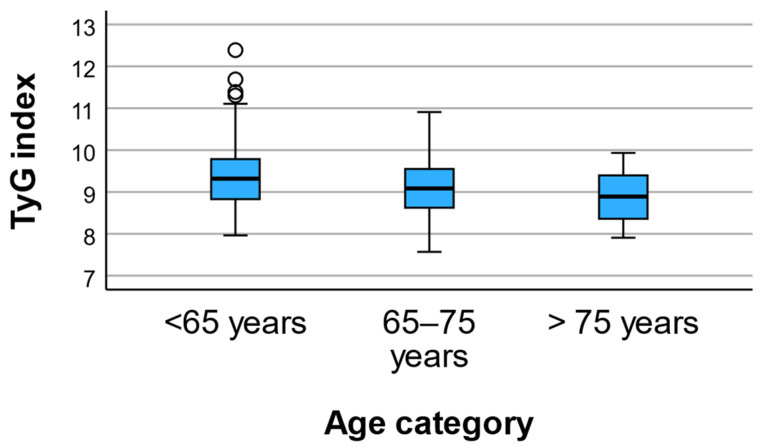
TyG index across age categories. Mean TyG index values decreased progressively across age groups, with the highest levels observed in participants aged <65 years and the lowest in those >75 years.

A similar age-related pattern was observed for lipid metabolism and markers of insulin resistance. Triglyceride concentrations decreased progressively across age strata, from 201.14 ± 182.82 mg/dL in individuals younger than 65 years to 146.65 ± 78.04 mg/dL and 125.07 ± 62.52 mg/dL in the intermediate and oldest age groups, respectively (*p* = 0.004). Consistently, the triglyceride–glucose (TyG) index declined significantly with age (9.43 ± 0.78 vs. 9.14 ± 0.61 vs. 8.88 ± 0.58; *p* = 0.001) (Figure 2). In contrast, HDL cholesterol levels did not differ significantly among age groups (*p* = 0.503).

### 3.4. Blood Pressure Profile

**Figure 3:**
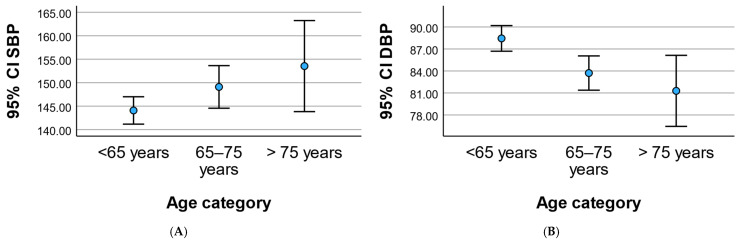
Age-related changes in blood pressure components. (**A**) Systolic blood pressure (SBP) and (**B**) diastolic blood pressure (DBP) across age categories. Data are presented as mean ± SD. *p* values were calculated using one-way ANOVA.

Age-related differences were observed in blood pressure components (Figure 3). Systolic blood pressure increased progressively across age categories, rising from 144.09 ± 19.57 mmHg in participants younger than 65 years to 149.10 ± 20.92 mmHg in those aged 65–75 years and reaching 153.54 ± 25.02 mmHg in individuals older than 75 years (*p* = 0.030). In contrast, diastolic blood pressure showed a significant decline with advancing age, decreasing from 88.44 ± 11.71 mmHg to 83.71 ± 10.77 mmHg and 81.29 ± 12.49 mmHg across the respective age groups (*p* = 0.001).

### 3.5. Hepatic and Hematological Markers

Significant age-related differences were observed in hepatic and hematological markers. Alanine aminotransferase (ALT) levels were highest in participants younger than 65 years and declined progressively with advancing age, decreasing from 33.71 ± 20.82 U/L to 21.39 ± 9.20 U/L and 19.00 ± 10.07 U/L in the 65–75 and >75 year age groups, respectively (*p* = 0.001). Aspartate aminotransferase (AST) showed a similar downward trend with age, although this association reached only borderline statistical significance (29.37 ± 25.60 vs. 22.50 ± 8.34 vs. 25.54 ± 19.81 U/L; *p* = 0.052).

Platelet count decreased significantly across age categories, from 250.09 ± 65.01 × 10^3^/µL in the youngest group to 238.10 ± 61.90 × 10^3^/µL in those aged 65–75 years and 212.89 ± 56.66 × 10^3^/µL in participants older than 75 years (*p* = 0.012). In contrast, the FIB-4 index increased markedly with age, rising from 1.18 ± 0.80 in individuals younger than 65 years to 1.57 ± 0.74 and 2.71 ± 3.12 in the intermediate and oldest age groups, respectively (*p* = 0.001).

### 3.6. Renal Function and Albuminuria

**Figure 4:**
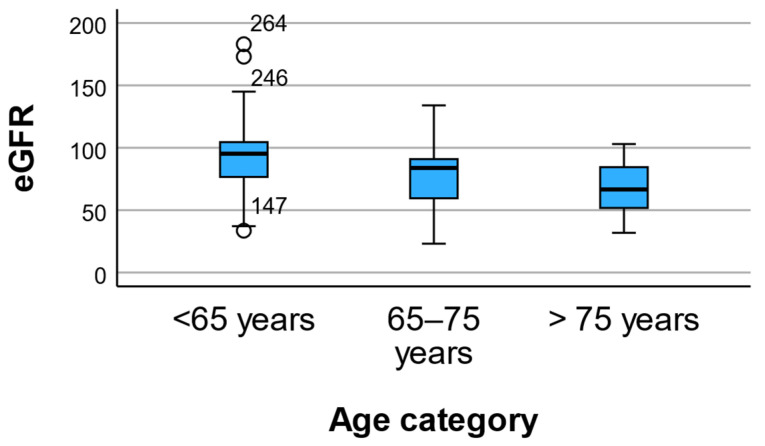
Estimated glomerular filtration rate (eGFR) across age categories. Data are presented as mean ± SD. Mean eGFR declined significantly with advancing age, indicating progressive age-related impairment of renal function. *p* values were calculated using one-way ANOVA.

Renal function parameters differed significantly across age categories (Figure 4). Serum creatinine levels increased with advancing age, rising from 0.82 ± 0.21 mg/dL in participants younger than 65 years to 0.92 ± 0.27 mg/dL in those aged 65–75 years and 0.91 ± 0.29 mg/dL in individuals older than 75 years (*p* = 0.005).

A pronounced age-related decline was observed in estimated glomerular filtration rate (eGFR), which decreased progressively from 91.31 ± 22.53 mL/min/1.73 m^2^ in the youngest group to 76.32 ± 20.34 mL/min/1.73 m^2^ and 67.73 ± 19.69 mL/min/1.73 m^2^ in the intermediate and oldest age groups, respectively (*p* = 0.001). In contrast, urinary albumin-to-creatinine ratio (UACR) showed no significant differences across age categories, despite substantial variability within groups (*p* = 0.243).

#### Sensitivity Analysis Using eGFR Categories

When renal function was analyzed using KDIGO eGFR categories rather than continuous values, a clear age gradient remained evident. Mean age category increased progressively from G1 (>90 mL/min/1.73 m^2^) to G3b (30–44 mL/min/1.73 m^2^). One-way ANOVA confirmed significant differences across categories (*F*(3283) = 13.31, *p* < 0.001).

The effect size was moderate-to-large (η^2^ = 0.124; ω^2^ = 0.114), indicating that the observed differences were clinically meaningful. Post hoc Bonferroni tests showed that individuals with preserved renal function (G1) were significantly younger than those in G2, G3a, and G3b categories (all *p* < 0.001).

These findings support that age-related differences in renal markers were not fully explained by physiological age-related decline in eGFR but reflect meaningful stratification across clinically relevant renal function categories.

### 3.7. Inflammatory Marker

C-reactive protein (CRP) levels did not differ significantly across age categories. Mean CRP values were 50.56 ± 74.69 mg/L in participants younger than 65 years, 57.37 ± 82.59 mg/L in those aged 65–75 years, and 39.83 ± 51.08 mg/L in individuals older than 75 years (*p* = 0.546), indicating no significant age-related differences in systemic inflammatory status.

### 3.8. Hierarchical Linear Regression Between Age Category, eGFR, and TyG Index

**Figure 5:**
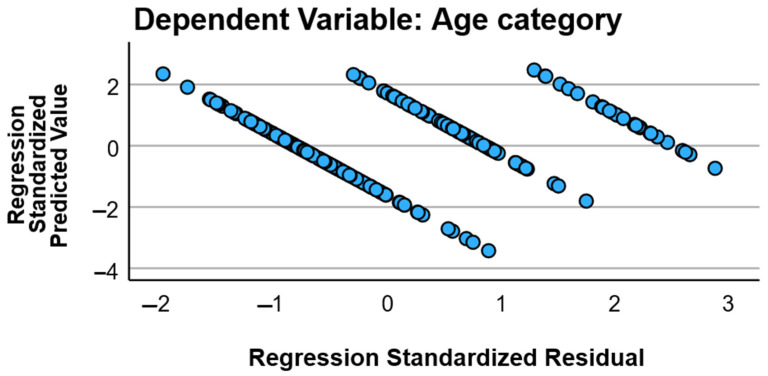
Standardized residuals versus standardized predicted values for the hierarchical regression model (dependent variable: age category). The scatterplot of standardized residuals against standardized predicted values was used to visually inspect model assumptions (linearity and homoscedasticity) and detect potential outliers. Most observations clustered within approximately ±3 standardized residual units, supporting acceptable residual dispersion for an exploratory linear model.

The distribution of the composite risk category score did not differ significantly between age groups (*p* = 0.882), despite marked age-related differences in individual cardio-metabolic and renal parameters (Figure 5). Results should be interpreted in terms of direction and association strength rather than prediction.

To further explore the joint relationship between renal function and insulin resistance across age strata, we performed a hierarchical multiple linear regression with age category as the dependent variable (coded ordinally, with higher values indicating older age), presented in Table 2. Predictors were entered in two steps using the Enter method: Model 1 included eGFR only, and Model 2 added the TyG index.

Model 1 (eGFR only) was statistically significant (*F*(1285) = 45.142, *p* < 0.001) and explained 13.7% of the variance in age category (*R*^2^ = 0.137; adjusted *R*^2^ = 0.134). Lower eGFR was associated with higher age category (B = −0.011, β = −0.370, *p* < 0.001).

After adding the TyG index (Model 2), the model remained significant (*F*(2284) = 35.328, *p* < 0.001) and explained 19.9% of the variance (*R*^2^ = 0.199; adjusted *R*^2^ = 0.194). The inclusion of the TyG index improved model fit by Δ*R*^2^ = 0.062, corresponding to an estimated F-change of ≈22.2 (*p* < 0.001). In Model 2, both predictors were independently associated with age category: eGFR remained negatively associated (B = −0.011, β = −0.367, *p* < 0.001) and the TyG index was also negatively associated (B = −0.227, β = −0.250, *p* < 0.001), indicating that higher insulin resistance (higher TyG) was more strongly represented in younger age categories.

Nonparametric analyses confirmed the main findings. Kruskal–Wallis tests showed significant age-group differences for triglycerides (*p* < 0.001) and fasting glucose (*p* = 0.038), while UACR and CRP remained non-significant.

## 4. Discussion

### 4.1. Cross-Sectional Age-Related Differences in Cardio–Reno–Metabolic Risk

In this cross-sectional analysis, we observed clear differences in dominant cardio–reno–metabolic risk patterns across age groups. Participants aged <65 years exhibited a predominantly metabolic phenotype, characterized by higher body mass index, waist circumference, fasting glucose, triglycerides, and TyG index. In contrast, older age groups—particularly those >75 years—demonstrated a shift toward a vascular–renal phenotype, marked by higher systolic blood pressure, lower diastolic blood pressure, and a progressive decline in estimated glomerular filtration rate.

This divergence suggests that aging is associated not merely with an accumulation of risk factors, but with a qualitative shift in the dominant pathophysiological pathways contributing to cardio–renal vulnerability. While metabolic dysregulation appears to be more prominent at younger ages, vascular stiffness, microvascular damage, and renal impairment become increasingly salient later in life [14,15,16,17]. These findings describe cross-sectional contrasts between age groups and should not be interpreted as evidence of individual-level temporal transitions. Detailed data on pharmacological treatment were not systematically available and may represent a source of residual confounding. Results should be interpreted in terms of direction and association strength rather than prediction.

### 4.2. Metabolic Burden at Younger Ages as a Potential Precursor of Vascular–Renal Injury

The elevated TyG index and triglyceride levels observed in the <65 age group indicate a higher degree of insulin resistance and metabolic stress. These alterations are known to promote endothelial dysfunction, low-grade inflammation, and early microvascular injury, even before overt clinical cardiovascular or renal disease becomes apparent.

Importantly, although markers of overt renal impairment (e.g., reduced eGFR) were more pronounced in older participants, the metabolic burden observed in younger individuals may represent an upstream driver of later vascular and renal damage. In this context, metabolic dysregulation at younger ages could be viewed as a risk amplifier, predisposing individuals to accelerated vascular aging and subsequent renal functional decline [17,18,19,20,21].

Thus, the apparent dissociation between metabolic risk at younger ages and vascular–renal manifestations at older ages may reflect different stages along a shared disease continuum rather than distinct, unrelated phenotypes.

### 4.3. Blood Pressure Pattern and Vascular Aging

The blood pressure profile observed across age groups provides additional insight into the vascular dimension of this risk reconfiguration. The progressive increase in systolic blood pressure combined with a decline in diastolic blood pressure is consistent with increased arterial stiffness and reduced vascular compliance in older adults.

This pattern has important clinical implications, as isolated systolic hypertension and widened pulse pressure are well-established predictors of cardiovascular events, cognitive decline, and renal damage in older populations. The emergence of this hemodynamic profile in the presence of declining eGFR underscores the close interplay between vascular aging and renal vulnerability [22,23,24].

### 4.4. Renal Function Decline as a Late Manifestation of Cumulative Risk

The marked decline in eGFR with advancing age observed in this study likely reflects the combined effects of chronological aging, vascular remodeling, and long-standing exposure to metabolic and hemodynamic stressors. Although the urinary albumin-to-creatinine ratio did not differ significantly between age groups, its wide variability suggests heterogeneity in microvascular renal involvement that may not be fully captured in a cross-sectional design.

From a clinical perspective, these findings reinforce the concept that reduced eGFR represents a relatively late marker of cumulative cardio–renal injury. Consequently, reliance solely on renal function indices at older ages may miss opportunities for earlier intervention during the metabolically driven phase of disease development [25,26,27]. The consistency of findings across both continuous and categorical renal measures strengthens confidence that the observed age-stratified patterns reflect genuine differences rather than index calibration effects.

### 4.5. Clinical Implications: Rationale for Early Vascular Risk Detection

Taken together, our findings support the notion that early detection of vascular and microvascular risk in metabolically burdened younger adults may be critical for preventing or delaying the transition toward overt vascular–renal disease in later life.

While younger individuals in this cohort exhibited a more adverse metabolic profile, they had not yet developed the pronounced vascular and renal alterations seen in older age groups. This window may represent a crucial opportunity for enhanced risk stratification, incorporating not only traditional metabolic markers but also early indicators of vascular dysfunction and renal stress [28,29,30].

Such an approach aligns with a life-course perspective on cardio–renal health, emphasizing prevention and early intervention rather than late-stage disease management [31].

### 4.6. Strengths and Limitations

The strengths of this study include the comprehensive assessment of metabolic, vascular, hepatic, and renal parameters across a broad age range and the use of clinically relevant composite indices such as the TyG index and eGFR.

However, several limitations warrant consideration. The cross-sectional design precludes causal inference and does not allow direct assessment of temporal risk progression. Potential confounding factors, including medication use, duration of metabolic disease, and survivor bias, may also influence age-related differences. Additionally, the FIB-4 index incorporates age into its calculation and should therefore be interpreted cautiously when comparing across age strata.

A key limitation relates to the age-dependent behavior of certain biomarkers. The estimated glomerular filtration rate physiologically declines with age even in the absence of overt kidney disease, and the FIB-4 index includes age as a direct component of its formula. Therefore, differences observed across age strata may partly reflect age-related calibration characteristics rather than purely pathological processes. Our findings should thus be interpreted as comparative risk patterns rather than diagnostic thresholds.

Information on pharmacological treatment was not systematically available and may represent a source of residual confounding.

Because age category is an ordinal outcome, linear regression treats the coding as approximately continuous. This approach is acceptable for exploratory modeling and effect direction; however, an ordinal logistic regression could be considered as a sensitivity analysis.

An additional limitation relates to the relatively small sample size in the oldest age group (>75 years, *n* = 28). This may reduce statistical precision and limit the generalizability of findings specific to this subgroup. Although the observed patterns were directionally consistent and clinically plausible, results concerning the oldest participants should be interpreted cautiously and confirmed in larger geriatric cohorts.

### 4.7. Future Directions

Longitudinal studies are needed to confirm whether the metabolic burden observed at younger ages directly contributes to subsequent vascular and renal deterioration. Future research should also explore whether early vascular or renal markers can improve risk prediction beyond traditional metabolic parameters and guide targeted preventive strategies across the aging continuum.

## 5. Conclusions

This study shows that cardio–reno–metabolic risk profiles differ across age groups. In contrast, older age groups—particularly those over 75 years—demonstrated a shift toward vascular–renal vulnerability, marked by higher systolic blood pressure, lower diastolic blood pressure, and a progressive decline in renal function.

These findings are consistent with the hypothesis that metabolic and vascular–renal markers may show different prominence across age strata, although longitudinal studies are required to confirm temporal sequencing. From a clinical perspective, this pattern underscores the importance of early identification of vascular and microvascular risk in metabolically burdened younger adults, before the onset of overt renal dysfunction and advanced vascular aging.

Although causal relationships cannot be established due to the cross-sectional design, our results support a life-course approach to cardio–renal risk assessment, emphasizing early prevention and timely intervention as key strategies to mitigate long-term vascular and renal complications across the aging continuum.

## Figures and Tables

**Table 1:** Clinical and biochemical characteristics of the study population according to age categories.

Variable	<65 Years (*n* = 175)	65–75 Years (*n* = 84)	>75 Years (*n* = 28)	*p*-Value (ANOVA)
Body mass index (kg/m^2^)	35.54 ± 5.03	35.04 ± 3.97	32.71 ± 2.29	0.010
Waist circumference (cm)	111.93 ± 10.82	111.25 ± 8.65	105.79 ± 4.75	0.009
Fasting plasma glucose (mg/dL)	162.48 ± 62.58	148.06 ± 44.51	133.00 ± 31.98	0.013
HDL cholesterol (mg/dL)	42.43 ± 9.58	41.74 ± 9.12	44.18 ± 10.54	0.503
Triglycerides (mg/dL)	201.14 ± 182.82	146.65 ± 78.04	125.07 ± 62.52	0.004
Systolic blood pressure (mmHg)	144.09 ± 19.57	149.10 ± 20.92	153.54 ± 25.02	0.031
Diastolic blood pressure (mmHg)	88.44 ± 11.71	83.71 ± 10.77	81.29 ± 12.49	<0.001
ALT (U/L)	33.71 ± 20.82	21.39 ± 9.20	19.00 ± 10.07	<0.001
AST (U/L)	29.37 ± 25.60	22.50 ± 8.34	25.54 ± 19.81	0.052
Platelet count (×10^3^/µL)	250.09 ± 65.01	238.10 ± 61.90	212.89 ± 56.66	0.012
C-reactive protein (mg/L)	50.56 ± 74.69	57.37 ± 82.59	39.83 ± 51.08	0.546
TyG index	9.43 ± 0.78	9.14 ± 0.61	8.88 ± 0.58	<0.001
FIB-4 index	1.18 ± 0.80	1.57 ± 0.74	2.71 ± 3.12	<0.001
Serum creatinine (mg/dL)	0.82 ± 0.21	0.92 ± 0.27	0.91 ± 0.29	0.005
eGFR (mL/min/1.73 m^2^)	91.31 ± 22.53	76.32 ± 20.34	67.73 ± 19.69	<0.001
UACR (mg/g)	30.28 ± 71.03	21.49 ± 44.34	37.50 ± 65.05	0.243
Risk category score	6.0 ± —	6.0 ± —	7.0 ± —	0.882

Data are presented as mean ± standard deviation. *p* values were calculated using one-way ANOVA for comparisons across age groups. ALT—alanine aminotransferase; AST—aspartate aminotransferase; CRP—C-reactive protein; eGFR—estimated glomerular filtration rate; TyG—triglyceride–glucose index; UACR—urinary albumin-to-creatinine ratio.

**Table 2:** Hierarchical linear regression models predicting age category from eGFR and TyG index.

Model	Predictors	*R*	*R* ^2^	Adjusted *R*^2^	SEE	B (Unstd.)	SE (B)	β (Std.)	t	*p*
1	Constant	0.370	0.137	0.134	0.62195	2.387	0.139	—	17.201	<0.001
	eGFR					−0.011	0.002	−0.370	−6.719	<0.001
2	Constant	0.446	0.199	0.194	0.60007	4.487	0.466	—	9.634	<0.001
	eGFR					−0.011	0.002	−0.367	−6.913	<0.001
	TyG index					−0.227	0.048	−0.250	−4.708	<0.001

Dependent variable: age category (ordinal coding, increasing with older age). SEE: standard error of the estimate. Model 1 predictors: eGFR. Model 2 predictors: eGFR + TyG index. Model 2 increased explained variance by Δ*R*^2^ = 0.062.

## Data Availability

The original data presented in the study are openly available in 10.6084/m9.figshare.30970342.

## Abbreviations

The following abbreviations are used in this manuscript:

ALT	Alanine aminotransferase
ANOVA	Analysis of variance
AST	Aspartate aminotransferase
BMI	Body mass index
BP	Blood pressure
CI	Confidence interval
CKD	Chronic kidney disease
CKD-EPI	Chronic Kidney Disease Epidemiology Collaboration
eGFR	Estimated glomerular filtration rate
FIB-4	Fibrosis-4 index
HDL-C	High-density lipoprotein cholesterol
HC3	Heteroscedasticity-consistent type 3
IQR	Interquartile range
MAFLD	Metabolic-associated fatty liver disease
SD	Standard deviation
SBP	Systolic blood pressure
TG	Triglycerides
TyG	Triglyceride–glucose index
UACR	Urinary albumin-to-creatinine ratio
